# Application of the Ultraleap 3Di-Based Gesture-Controlled 3D Imaging Visualization System in Pulmonary Segmentectomy: A Single-Center Prospective Study

**DOI:** 10.3390/bioengineering13030284

**Published:** 2026-02-28

**Authors:** Zhengnan Liu, Bin Wang, Chengrun Li, Ruiji Chen, Jixing Lin, Jie Li

**Affiliations:** 1 Chinese PLA Medical School, Beijing 100853, China; 18514557889@163.com; 2 Department of Thoracic Surgery, The First Medical Center, Chinese PLA General Hospital, Beijing 100853, China; banxy0119@163.com (B.W.); lichengrun@yeah.net (C.L.); 3 Department of Thoracic Surgery, Hainan Hospital of Chinese PLA General Hospital, Sanya 572013, China; rickychan0225@163.com (R.C.); linjixing301@sina.com (J.L.)

**Keywords:** gesture control, non-contact interaction, segmentectomy, lung cancer

## Abstract

Objective: Pulmonary segmentectomy serves as a crucial approach for treating early-stage lung cancer. However, this procedure demands precise identification of segmental anatomy, and surgeons often need to repeatedly consult the patient’s 3D imaging data or other medical records during the operation. Traditional contact-based intraoperative imaging assistance devices involve cumbersome operation and pose risks to the sterile environment. This study aims to evaluate the clinical utility of an Ultraleap 3Di-based gesture-controlled 3D imaging visualization system for non-contact interaction during pulmonary segmentectomy in patients with early-stage lung cancer. Methods: This study enrolled 58 patients with early-stage non-small cell lung cancer scheduled for video-assisted thoracoscopic pulmonary segmentectomy from June 2025 to December 2025. Participants were randomly assigned to either the experimental group or the control group. Intraoperatively, the experimental group utilized the Ultraleap 3Di system for non-contact 3D image review, while the control group relied on conventional contact-based devices for image retrieval, which was operated by non-sterile assistants. The compared outcomes included intraoperative image retrieval time, total operative time, intraoperative blood loss, R0 resection rate, postoperative drainage duration, and surgeon satisfaction. Results: The baseline characteristics were comparable between the two groups. The mean age was 53.66 ± 9.12 years in the experimental group and 55.21 ± 8.76 years in the control group (t = −0.66, *p* > 0.05); the experimental group included 16 males and 13 females, while the control group included 14 males and 15 females (χ^2^ = 0.276, *p* > 0.05). Preoperative pulmonary function, as measured by FEV1/FVC ratio, was 74.48 ± 4.75% in the experimental group versus 76.08 ± 4.51% in the control group (t = −1.31, *p* > 0.05). The image retrieval time in the experimental group was significantly shorter than that in the control group (75.16 ± 19.38 s versus 209.59 ± 28.13 s, t = −21.19, *p* < 0.001, 95% CI [−147.13, −121.72], Cohen’s d = −5.57). The total operative time was also reduced (88.72 ± 13.82 min versus 96.55 ± 13.90 min, t = −2.15, *p* = 0.036, 95% CI [−15.12, −0.53], Cohen’s d = −0.57). No significant differences were observed between the two groups in terms of R0 resection rate (both 100%), intraoperative blood loss, or postoperative drainage duration (*p* > 0.05). The operating surgeons rated the system highly for image clarity, navigation timeliness, and overall utility, while the score for operational convenience was relatively neutral (mean score 3.2). Conclusions: The Ultraleap 3Di-based non-contact visualization system reduces the time required for intraoperative image retrieval and improves overall procedural efficiency in segmentectomy, without compromising surgical safety or oncological radicality. Future efforts should focus on optimizing the intuitiveness of gesture interaction and exploring its integration with augmented reality and artificial intelligence to further advance the system’s intelligence and practical utility.

## 1. Introduction

Recent global cancer statistics indicate that lung cancer ranks as the most commonly diagnosed cancer worldwide, with approximately 2.5 million new cases annually—accounting for one-eighth of all new cancer cases globally [1]. With the widespread adoption of low-dose spiral CT screening, the detection rate of early-stage lung cancer has increased significantly. Patients with early-stage disease achieve a 5-year survival rate of 70–90%, substantially higher than those diagnosed at advanced stages [2]. For early-stage non-small cell lung cancer (NSCLC), surgical resection remains the cornerstone of curative treatment. While lobectomy was historically the standard procedure, the indications and techniques for segmentectomy have evolved with advances in surgical technology and a deeper understanding of lung cancer biology [3]. Consistent with recent clinical evidence, anatomical segmentectomy is now recommended for patients with early-stage NSCLC, particularly those with tumor diameters ≤2 cm and a consolidation-to-tumor ratio >0.5, due to its favorable survival outcomes and superior lung function preservation [4,5,6]. However, segmentectomy poses significantly greater technical challenges than lobectomy, as its successful execution heavily relies on the surgeon’s precise understanding of the pulmonary segmental anatomy. The anatomical boundaries of a segment are defined collectively by the intersegmental veins, segmental bronchi, and segmental arteries. Notably, substantial individual variations in this anatomy exist among patients—including instances of absent intersegmental veins or variant arterial branching. It has been reported that approximately 12.3% of patients exhibit variations in their pulmonary venous anatomy [7]. Intraoperative failure to clearly delineate these structures risks residual disease, vascular injury, and excessive pulmonary function loss, compromising patient outcomes. During pulmonary segmentectomy, surgeons must repeatedly consult preoperative 3D reconstructions or intraoperative CT scans to verify anatomical boundaries. Real-time visualization and interactive control of pulmonary segmental anatomy via intraoperative imaging are therefore critical to enhancing surgical precision and safety.

In recent years, with the rapid advancement of three-dimensional imaging technology, the application of 3D printing in the medical field has gradually transitioned from experimentation to clinical practice [8], demonstrating unique value particularly in thoracic pulmonary segmentectomy. In addition, 3D-printed models based on high-resolution CT data can physically and intuitively present the complex pulmonary segmental vessels, bronchi [9], and nodular structures, offering new solutions for preoperative planning, anatomical education, and intraoperative reference. Currently, intraoperative imaging assistance in clinical practice primarily relies on conventional contact devices (e.g., mouse, keyboard, touchscreen) for operation. In the contemporary operating room, retrieving images from the medical e-imaging system primarily entails the surgeon requesting assistance from off-table personnel, such as circulating nurses or anesthesiologists. However, due to the frequent lack of experience among non-sterile personnel in operating imaging systems, misoperations often occur. This situation may even require the surgeon or assistant to leave the sterile field to operate the computer system directly, followed by re-sterilization before returning to the procedure. Consequently, the surgical workflow can be severely disrupted [10].

To address the limitations of conventional contact-based assistance systems, non-contact gesture control technology has seen rapid development in the surgical field in recent years. Although early gesture control devices such as Kinect and Leap Motion have been trialed in surgery [11,12,13], they possess notable technical shortcomings: Kinect offers a relatively narrow field of view (57° × 43°), insufficient to cover the operational space around the surgical table, and its low frame rate (30 fps) makes it prone to recognition latency; Leap Motion, conversely, has a restricted tracking range (0–60 cm), requiring surgeons to operate in close proximity—a constraint incompatible with standard surgical practice—and its performance is highly sensitive to ambient lighting, leading to significantly reduced recognition accuracy under the strong lights or shadows typical of operating rooms.

Based on the aforementioned technical challenges, our team previously developed a non-contact gesture-controlled 3D imaging visualization system based on the Ultraleap 3Di stereo hand-tracking camera, which underwent preliminary validation in a simulated surgical environment [14]. However, significant discrepancies exist between simulated conditions and actual clinical settings—such as the absence of interference from patient physiological movements, obstruction by surgical instruments, and staff movement—raising questions about the system’s efficacy in real-world pulmonary segmentectomy procedures. Therefore, we conducted a clinical trial involving 58 cases of early-stage lung cancer patients undergoing segmentectomy, aiming to further evaluate the clinical value of the system and provide evidence for its technological translation.

## 2. Materials and Methods

This clinical trial was conducted in accordance with the ethical principles of the Declaration of Helsinki. The study was carried out within the Department of Thoracic Surgery at the First Medical Center of the Chinese PLA General Hospital and received approval from the hospital’s Institutional Review Board (approval number: S2025-1024-01).

### 2.1. Study Population and Eligibility Criteria

This study enrolled 58 patients with early-stage non-small cell lung cancer scheduled for thoracoscopic segmentectomy between June 2025 and December 2025. The inclusion criteria were as follows: (I) confirmed early-stage NSCLC with lesions confined to specific pulmonary segments; (II) chest CT showing a peripherally located tumor with a diameter of ≤2 cm; (III) age between 18 and 75 years; (IV) absence of severe cardiovascular, hepatic, or renal dysfunction; (V) provision of informed consent to participate in the study. Exclusion criteria were as follows: (I) patients requiring lobectomy for the tumor, or those with ground-glass nodules < 2 cm amenable to wedge resection; (II) presence of severe dysfunction of major organs (e.g., heart, liver, kidney) or coagulation disorders; (III) unwillingness to participate in the trial.

This study employed a parallel, two-arm, randomized controlled design. The randomization sequence was generated using IBM SPSS Statistics 26 by an independent staff member not involved in patient recruitment, surgery, or outcome assessment. Participants were allocated to either the experimental group or the control group at a 1:1 ratio. To ensure allocation concealment and prevent selection bias, the assignment results were placed in sequentially numbered, sealed, opaque envelopes. After obtaining written informed consent and formal enrollment, the same independent staff member opened the corresponding envelope in sequence before surgery and notified the surgical team of the group allocation.

All surgical procedures in this study were performed by four attending surgeons, each with over five years of experience in thoracoscopic surgery (hereinafter referred to as “Surgeon A”, “Surgeon B”, “Surgeon C”, and “Surgeon D”, respectively). All four surgeons were involved throughout both the experimental and control group surgeries and had uniformly watched an operation demonstration video of the gesture-controlled system before the start of the experiment.

To control for the influence of individual surgeon differences on the study results, a balanced design was implemented for the distribution of cases among the four surgeons across the two groups. Surgeries in both the experimental and control groups were sequentially divided into three phases according to the operative sequence: early phase (cases 1–10), middle phase (cases 11–20), and late phase (cases 21–29). The four surgeons participated in a balanced distribution of cases across each phase, as follows: Surgeon A completed 3, 3, and 2 cases in the early, middle, and late phases, respectively; Surgeon B completed 2, 3, and 2 cases; Surgeon C completed 2, 2, and 3 cases; and Surgeon D completed 3, 2, and 2 cases.

### 2.2. Device Architecture

**Figure 1:**
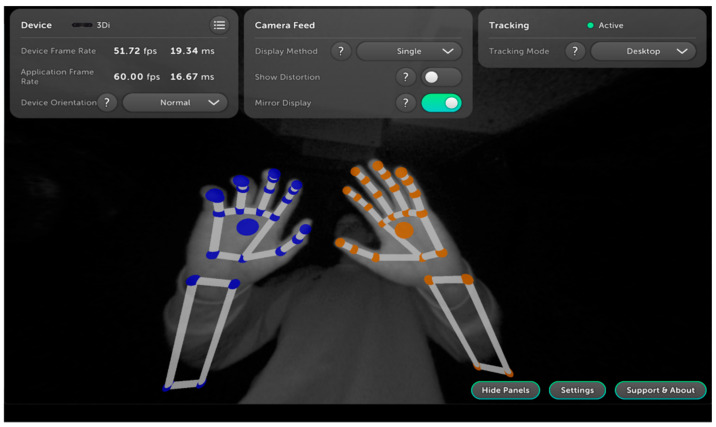
Gesture recognition modes and states.

The system was developed based on Ultraleap 3Di stereoscopic hand-tracking technology and comprises two main components within the interaction module: hand tracking and gesture recognition. The tracking subsystem is primarily constructed with two infrared cameras and an 850 nm infrared LED light source. For gesture recognition, the system employs a combined “Convolutional Neural Network–Track Before Detect (CNN-TBD)” framework. First, the binocular infrared cameras capture images of the surgeon’s hands. These images undergo denoising and preprocessing before being fed into a CNN for hand feature extraction. Subsequently, the TBD algorithm traces the dynamic trajectories of 21 hand key points, where energy accumulation mitigates inter-frame jitter and noise to ensure gesture recognition continuity. Finally, the system maps the recognized gestures to predefined commands (Table 1) and executes the corresponding operations in real time (Figure 1).

### 2.3. Surgical Procedure

**Figure 2:**
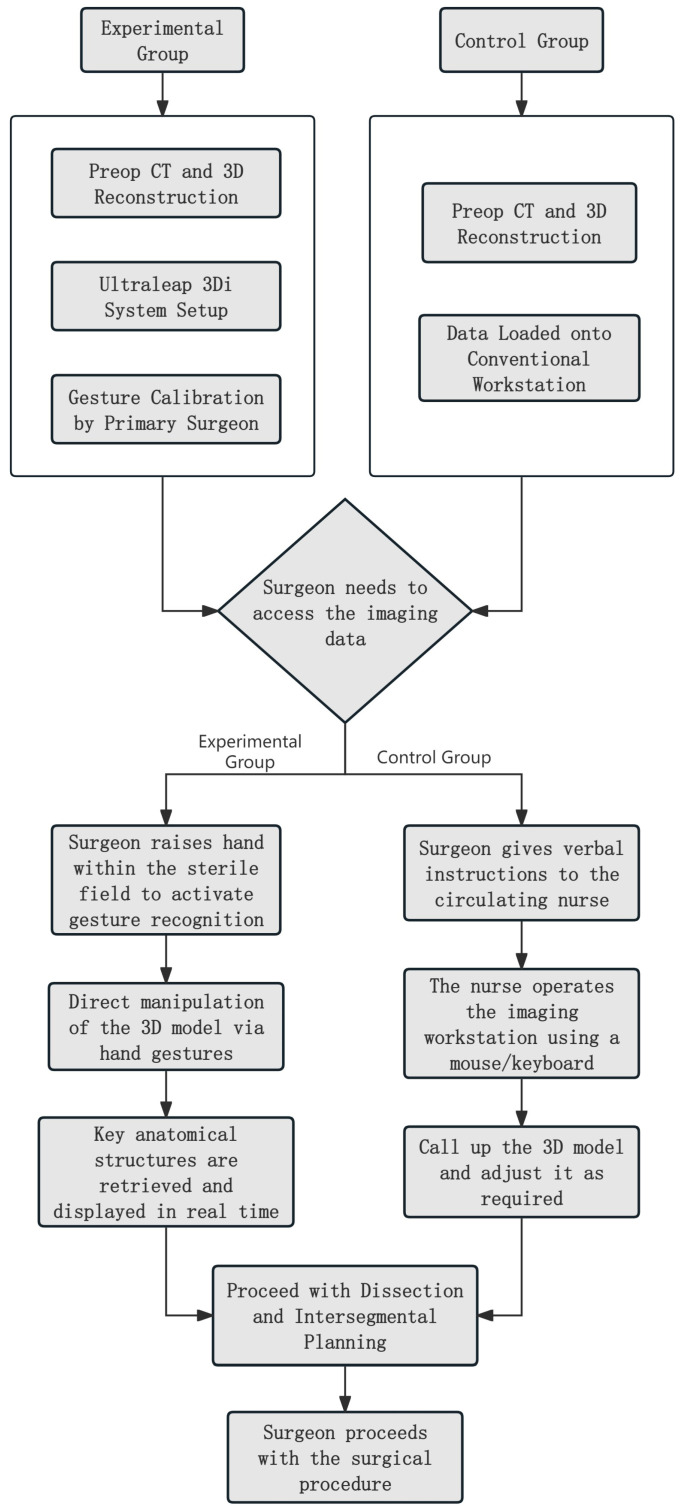
Surgical procedure flow chart of the experimental group and control group.

The surgical procedures for both groups strictly adhered to the standardized segmentectomy protocols. The core distinction between the two groups lay in the method of intraoperative imaging assistance employed. The surgical flowcharts for the experimental group and the control group are shown in Figure 2.

#### 2.3.1. Preparation Stage

**Figure 3:**
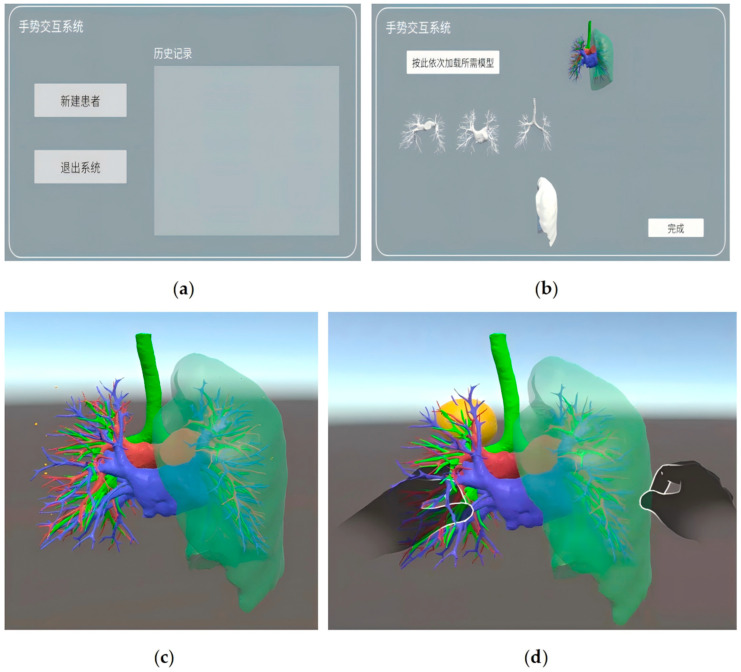
Hand Control View software interface panels. (**a**) This figure shows the main interface of the system, where the “New Patient” button is used to select and import patient lung structures. (**b**) The model loading interface permits preoperative import of discrete anatomical structures—including pulmonary arteries, veins, bronchi, and nodules—for intraoperative visualization. (**c**,**d**) Upon clicking the completion button, the system enters the control interface. Virtual 3D hands appear on the display when the surgeon’s hands enter the operational zone (~45 cm above the sensor). The sensor recognizes gestures in real time, recording three-dimensional hand positions within its coordinate frame.

All patients underwent preoperative contrast-enhanced chest CT scans. The imaging data were imported into a three-dimensional reconstruction workstation to generate precise models of pulmonary vessels, bronchi, and the target nodule. For the experimental group, the Ultraleap 3Di system was set up one hour before surgery. This involved connecting the Ultraleap 3Di camera to a personal computer and positioning it beside the operating table (approximately 1.2 m away, ensuring its field of view covered the surgeon’s hand movement area). The patient’s 3D reconstructed model, incorporating anatomical structures such as the pulmonary arteries, pulmonary veins, bronchus, and pulmonary nodule, was then loaded into the “Hand Control View v1” software (Figure 3), with model brightness and contrast adjusted for optimal surgical planning and intraoperative clarity. The Ultraleap 3Di system undergoes initial calibration at the factory and typically requires no recalibration under normal operating conditions. To further enhance operational precision, however, we performed a gesture recalibration whenever the primary surgeon changed. The calibration procedure was as follows: wearing sterile gloves, the surgeon performed each of the five basic gestures three times following on-screen prompts, allowing the system to automatically adapt to their hand dimensions and operational habits. The entire process was completed in approximately two minutes. In the control group, patient imaging data was stored on a conventional medical imaging workstation in the operating room.

#### 2.3.2. Operational Stage

Following the induction of general anesthesia, two ports were established at the 4th intercostal space on the anterior axillary line and the 7th intercostal space on the midaxillary line. A Storz 4K ultra-high-definition thoracoscopic system (Karl Storz Endoscopy, Shanghai, China) with a 30° lens was used to create the thoracoscopic cavity. The procedure commenced with a thorough exploration of the thoracic cavity to assess lung mobility, identify any adhesions, and locate the approximate position of the lesion. Subsequently, the surgery progressed to the hilar dissection phase, at which point a significant divergence in the method of image retrieval emerged between the two study groups.

During dissection of segmental vessels and bronchi, as well as during intersegmental plane planning and division, the surgeon in the experimental group could naturally raise a hand within the sterile field to activate the gesture capture function and directly retrieve the 3D model in real time via the gesture control system. For instance, while managing segmental arteries, the “transparency adjustment” gesture was used to hide certain pulmonary veins, thereby clearly exposing deeper arterial branches and their anatomical variations. Before dissecting segmental bronchi, the “rotation” and “scaling” gestures enabled multi-angle inspection of the spatial relationship between bronchi and adjacent vessels to prevent inadvertent injury. When planning the intersegmental plane, the surgeon employed the bimanual “thumb–index finger pinch” gesture, pulling both hands outward to zoom in on the model for detailed observation of intersegmental venous pathways. Using the “translation” gesture, the model was aligned with the thoracoscopic view. The “transparency adjustment” function was then employed to progressively peel away the pulmonary parenchyma, leaving only the intersegmental veins as anatomical landmarks to guide the dissection path for the linear stapler. The entire image retrieval process required no assistance from others outside the operating table.

In the control group, the surgeon relied entirely on conventional contact-based devices to retrieve and manipulate 3D models. When intraoperative reference to a 3D reconstruction was required, the surgeon had to verbally instruct the circulating nurse to perform specific tasks, such as “Please magnify the branch of the pulmonary artery,” “Rotate to the coronal plane,” or “Hide the bronchial structures.” Due to the nurse’s limited understanding of anatomical details, repeated clarification was often necessary.

Subsequent core resection procedures were followed identically in both groups.

### 2.4. Observation Indicators

To comprehensively evaluate the clinical value of the system, this study established baseline indicators, primary observation indicators, and secondary observation indicators. All measurements were performed by personnel not involved in the surgical procedures to avoid measurement bias.

#### 2.4.1. Baseline Indicators

To ensure the scientific validity and comparability of our findings, we collected several baseline variables that could potentially influence surgical outcomes. Demographic characteristics, including patient age and sex, were recorded. For the assessment of pulmonary function, the ratio of forced expiratory volume in 1 s to forced vital capacity (FEV1/FVC) was used as the key metric.

#### 2.4.2. Primary Observation Indicators

The primary observation indicators focused on core indicators of surgical efficiency and safety, including image retrieval time, total operative time, and intraoperative blood loss, all recorded in real time during surgery. Image retrieval time was measured by the circulating nurse using a calibrated electronic stopwatch (accurate to 0.01 s). In the experimental group, timing commenced when the operating surgeon first raised a hand to activate the Ultraleap 3Di system (indicated by the appearance of virtual hands on the screen) and ceased when the surgeon had clearly identified all key anatomical boundaries of the target pulmonary segment—including the courses and variant structures of the target segmental arteries, veins, and bronchi—via gesture control, and verbally confirmed the identification. In the control group, timing began when the operating surgeon issued a command to the off-table personnel for image retrieval and ended when the aforementioned anatomical boundaries were clearly identified and verbally confirmed by the surgeon. Total operative time was defined as the continuous duration from skin incision to the closure of the final surgical port, meticulously monitored and recorded by the circulating nurse using the operating room timing system. Intraoperative blood loss was quantified via a combined method employing precise suction measurement and weighed gauze calculation.

#### 2.4.3. Secondary Observation Indicators

Secondary observation indicators included the R0 resection rate, postoperative drainage duration, and the operator satisfaction score. The R0 resection rate was defined as the proportion of surgeries in which the tumor was completely removed with negative microscopic margins. Postoperative drainage duration referred to the time from chest tube insertion to its removal, with removal criteria being a 24 h drainage volume of <200 mL, absence of air leakage, and satisfactory lung re-expansion on chest X-ray. Additionally, within 24 h after completing their respective surgeries, the four operating surgeons filled out a Likert 5-point satisfaction scale (5 = Very good, 4 = Good, 3 = Average, 2 = Poor, and 1 = Very poor). The evaluation dimensions included operational convenience, image clarity, navigation timeliness, and overall practicality. After aggregating the data from all 29 cases in the experimental group, the mean score for each dimension was calculated.

## 3. Results

Following data collection, all statistical analyses were carried out using IBM SPSS Statistics 26.

### 3.1. Comparison of Baseline Indicators

The two patient groups demonstrated comparable baseline indicators, with no statistically significant differences in gender, age, or pulmonary function status (Table 2). Regarding demographic features, the mean age of patients in the experimental group was 53.66 ± 9.12 years, compared to 55.21 ± 8.76 years in the control group. An independent samples t-test confirmed no significant difference in age distribution between the groups (t = −0.66, *p* > 0.05). In terms of gender composition, the experimental group comprised 16 males and 13 females, while the control group included 14 males and 15 females. A Chi-square test indicated a balanced gender ratio across the groups (χ^2^ = 0.276, *p* > 0.05). Preoperative pulmonary function tests showed an FEV1/FVC ratio of 74.48 ± 4.75% in the experimental group and 76.08 ± 4.51% in the control group, indicating adequate pulmonary function reserve in both cohorts with no significant intergroup difference (t = −1.31, *p* > 0.05).

### 3.2. Comparison of Primary Observation Indicators

Analysis of the primary outcomes (Table 3) revealed that the experimental group demonstrated superior performance over the control group in both image retrieval time and total operation time. Specifically, the image retrieval time in the experimental group (75.16 ± 19.38 s) was significantly shorter than that in the control group (209.59 ± 28.13 s), with a statistically significant difference (t = −21.19, *p* < 0.001, 95% CI [−147.13, −121.72], Cohen’s d = −5.57).

The between-group difference in total operative time further underscores the efficiency advantage of the system. The mean operative time was shorter in the experimental group (88.72 ± 13.82 min) than in the control group (96.55 ± 13.90 min; t = −2.15, *p* = 0.036, 95% CI [−15.12, −0.53], Cohen’s d = −0.57). This reduction was primarily attributed to the enhanced image retrieval efficiency, which minimized non-productive waiting during surgery. Furthermore, the precise interaction with the three-dimensional model simplified key procedural steps—such as vascular dissection and intersegmental plane planning—by providing clear anatomical visualization. This clarity eliminated the need for repeated exploration due to ambiguous anatomy, thereby streamlining the overall surgical procedure.

Regarding intraoperative blood loss, no significant difference was observed between the experimental group (26.21 ± 12.37 mL) and the control group (29.31 ± 14.13 mL; t = −0.89, *p* = 0.38, 95% CI [−10.09, 3.88], Cohen’s d = −0.23). This result indicates that the Ultraleap 3Di-based non-contact visualization system improved surgical efficiency without increasing the risk of surgical bleeding.

### 3.3. Comparison of Secondary Observation Indicators

Results for the secondary observation indicators are summarized in Table 4. Regarding oncological radicality, the R0 resection rate was 100% in both the experimental and control groups, indicating that both achieved the standard of oncological completeness for segmentectomy. This finding demonstrates that the Ultraleap 3Di system enhanced surgical efficiency without compromising the thoroughness of resection. For postoperative recovery indicators, the two groups exhibited homogeneous profiles: the postoperative drainage duration was 2.41 ± 0.73 days in the experimental group versus 2.28 ± 0.53 days in the control group (t = 0.82, *p* = 0.41, 95% CI [−0.20, 0.47], Cohen’s d = 0.22). This result further indicates that the system did not alter the natural course of postoperative recovery.

**Figure 4:**
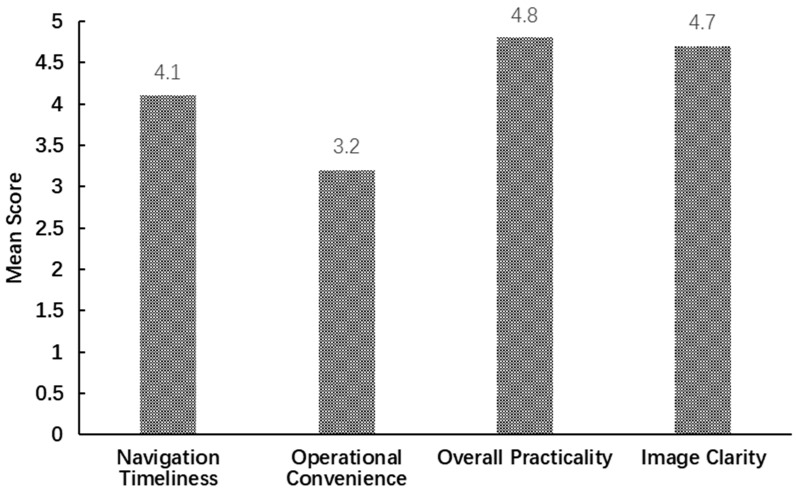
Surgeon satisfaction ratings for the gesture-controlled system.

Following the surgical procedures, the operating surgeon evaluated the system across four predefined dimensions. The average scores are presented in Figure 4: operational convenience scored 3.2, overall practicality scored 4.8, navigation timeliness scored 4.1, and image clarity scored 4.7. These results indicate that the system received high recognition for its image quality and core functionality, while there remains room for improvement in the operational experience. Specifically, the near-perfect scores in image clarity and overall practicality demonstrate that the system provides detailed and anatomically realistic 3D models, which accurately represent segmental anatomy. Furthermore, its non-contact interaction concept effectively addresses the critical intraoperative concern of maintaining sterility. Navigation timeliness was also favorably evaluated, reflecting the Ultraleap 3Di system can adequately support the need for real-time intraoperative decision-making. In contrast, operational convenience received relatively neutral ratings.

The gap between the score for operational convenience and overall practicality reflects the persistent issue of “cognitive friction” in clinical applications. To investigate whether this phenomenon is correlated with a learning curve, we analyzed the three phases of the 29 cases in the experimental group. The mean image retrieval time and standard deviation for each phase were as follows: early phase 90.07 ± 17.41 s, middle phase 72.98 ± 16.36 s, and late phase 61.02 ± 12.82 s. After normality testing and homogeneity of variance testing, a one-way analysis of variance (ANOVA) was further employed to compare the differences in image retrieval time across the phases, with a significance level set at α = 0.05. The results showed a significant difference in image retrieval time among the three phases, F(2,26) = 8.20, *p* = 0.002. Further post hoc multiple comparisons revealed that the image retrieval time in the early phase was significantly longer than that in the late phase (mean difference = 29.04 s, *p* = 0.001, 95% CI [10.52, 47.56]). No statistically significant differences were observed between the early and middle phases, nor between the middle and late phases. These findings indicate that image retrieval time tended to decrease with increasing surgical experience, with a significant learning effect primarily observed from the early to the late phase, suggesting that improved proficiency with the system contributes to reduced image retrieval time. However, the absence of a significant difference between the middle and late phases may suggest that the rapid ascent phase of the learning curve is primarily concentrated within the first 20 cases. Additionally, some surgeons reported that complex gestures requiring bimanual coordination, such as scaling, were occasionally not fluid enough in high-stress surgical settings. This indicates that although the learning curve has improved operational efficiency to some extent, future efforts should focus on further optimizing the gesture recognition algorithm to enhance the system’s robustness in complex surgical environments, as well as exploring customizable gesture configurations to reduce the cognitive burden on the surgeon.

## 4. Discussion

This single-center clinical application study, building upon prior validation in a simulated operating room environment, further confirms the clinical value of the Ultraleap 3Di-based gesture-controlled 3D imaging visualization system in segmentectomy for early-stage lung cancer. Our findings demonstrate that the experimental group exhibited shorter intraoperative image retrieval times and total operative times compared to the control group. This efficiency advantage is attributed to the system’s ability to consolidate the traditionally fragmented, multi-step process of image retrieval into a seamless and continuous decision-making support workflow. The high satisfaction ratings from the operating surgeons provide valuable iterative insights for future system refinement. The score for operational convenience revealed certain limitations during the early developmental phase of the technology. Subsequent efforts could focus on developing more intelligent deep learning techniques capable of recognizing a wider range of gestures and increasing the degrees of freedom in gesture operations, thereby enabling more flexible touchless visualization. From a hardware perspective, employing lightweight neural networks (e.g., MobileNetV4) and edge computing devices could help reduce recognition latency.

Although the total operative time in the experimental group was shortened by only approximately 8 min, the control group in this study relied on a model where non-sterile personnel outside the sterile field assisted with image retrieval. If the non-sterile personnel failed to accurately comprehend the operating surgeon’s anatomical instructions, the surgeon often had to remove the surgical gown, personally operate the imaging workstation, and then undergo re-scrubbing and re-gowning before returning to the operating table. This process typically requires about 10 min, which would further widen the efficiency gap between the two groups.

Notably, the core focus of surgical intelligent navigation is shifting from “static visualization” to “dynamic intelligence,” a trend that offers a clear pathway for this system’s evolution. Future iterations could integrate gesture recognition with augmented reality [15] or artificial intelligence [16] to further enhance its applicability and functionality in the operating room. Furthermore, the CNN-TBD architecture can be integrated with a U-Net segmentation model to achieve a fully automated workflow of “gesture-based activation—automatic recognition—variation alert”. Specifically, when the surgeon focuses on the target lung segment via gestures, the system could automatically identify arterial branch variations, highlight them in red, and simultaneously display management strategies from historical cases with similar anatomy. This dual-driven approach, combining technical assistance with empirical wisdom, holds promise for further reducing the learning curve for junior surgeons.

The application of multimodal image fusion technology holds the potential to further expand the functional boundaries of the current system. While the existing system relies on preoperative CT-based 3D reconstruction, intraoperative factors such as lung collapse and vascular pulsation may lead to discrepancies between the displayed images and the actual surgical field. Extensive prior research has demonstrated that fluorescence-assisted thoracoscopy significantly reduces operative time and the incidence of air leakage during identification of intersegmental planes and dissection of vessels, without increasing postoperative complications [17,18]. The proposed system can adopt this approach by integrating near-infrared fluorescence thoracoscopic imaging through expanded hardware interfaces. Surgeons can seamlessly switch between single-modality and fusion-mode displays using a simple “two-finger swipe” gesture. For instance, the CT-based 3D model ensures precision during vascular dissection, while the fluorescence fusion imaging clarifies boundaries during intersegmental plane division. This dynamic adaptability effectively addresses the dual challenges of “anatomical localization” and “functional demarcation” encountered in pulmonary segmentectomy.

## 5. Limitations

Although this study confirms the advantages of the gesture-controlled system in improving surgical efficiency, we must confront a core technical challenge: the discrepancy between static preoperative imaging and dynamic intraoperative anatomy. Unlike static organs, the lung is a soft organ that undergoes continuous, large-scale, nonlinear deformation during surgery. Under conditions of intraoperative lung collapse, the preoperative CT images do not perfectly match the real-time intraoperative anatomy, and the positional and morphological relationships of vessels, bronchi, and nodules undergo significant changes. In this study, surgeons primarily relied on spatial imagination and extensive anatomical experience to compensate for this gap. This navigation approach, driven by experience rather than real-time data, limits the system’s generalizability in cases involving complex anatomical variations or among junior surgeons. Future efforts should address the challenges of deformation prediction and registration under intraoperative lung collapse conditions.

In addition, this study has a relatively limited sample size, and all cases were sourced from a single medical institution, which may constrain the external validity of the findings. Variations in surgical workflows, equipment configurations, and team coordination patterns across different institutions necessitate further validation of the system’s generalizability in complex clinical environments through multi-center, large-sample cohorts. Secondly, while the study primarily focused on intraoperative efficiency metrics and short-term postoperative recovery parameters, it did not assess long-term oncological outcomes or the preservation of lung function over time. Furthermore, this study did not systematically record other clinical variables that might influence total operative time and the frequency of image retrieval, such as the location of the target pulmonary segment, the complexity of the surgical procedure (e.g., simple vs. complex segmentectomy), and the degree of fissure development. Future studies should incorporate a more comprehensive set of preoperative assessment indicators.

Lastly, this study has inherent risks of performance bias and measurement bias. The experimental group utilized the Ultraleap 3Di-based non-contact gesture-controlled system, while the control group relied on conventional contact-based imaging assistance. Given the substantial differences between the two intraoperative interventions, blinding of the surgeons was not feasible. The surgeons’ awareness of group allocation may have influenced their intraoperative behaviors. For instance, they may have been more motivated to demonstrate the efficacy of the novel system in the experimental group, thereby introducing performance bias. Furthermore, outcome assessors were also aware of group assignments when recording indicators such as image retrieval time and operative time. This awareness posed a potential risk of measurement bias.

## 6. Conclusions

The Ultraleap 3Di-based non-contact gesture-controlled 3D imaging visualization system demonstrates significant clinical efficacy in segmentectomy for early-stage lung cancer, offering a more efficient intraoperative image retrieval solution for precise pulmonary segmental resection. To further unlock the clinical potential of this technology, however, it is essential to establish an innovation cycle driven jointly by engineering optimization and evidence-based medical validation. Future studies should focus on conducting multicenter, multi-procedural, and stratified clinical cohort investigations to systematically evaluate the impact of anatomical complexity, surgeon experience gradient, and operating room environments on system performance, thereby laying a solid foundation for its integration into the future intelligent surgical ecosystem.

## Figures and Tables

**Table 1:** Mapping of hand gestures to system functions.

Function Type	Gesture Description	Operation Logic
Selection	Index finger points vertically at the target, other fingers closed. Activated by an “air-push” gesture (moving finger towards the display).	“Index finger point” locates the target structure. The “air-push” is equivalent to a “mouse click”.
Rotation	Thumb and index finger pinched together (other three fingers naturally extended). Rotate wrist up, down, left, or right.	The model rotates in the same direction as the wrist rotation (e.g., wrist up rotates model up).
Translation	Thumb and index finger pinched together (other three fingers naturally extended). Move hand up, down, left, or right.	The model pans in the same direction as the hand movement.
Scaling	Both hands with thumb and index fingers pinched together (other fingers naturally extended). Move both hands simultaneously outward or inward.	Hands moving outward: model zooms in; inward: model zooms out
Transparency adjustment	Point at the target structure with one finger, then perform “air-push” 1–2 times.	First push: decreases target structure transparency by 50%; second push: completely hides structure

**Table 2:** Comparison of baseline indicators between the two groups.

Outcome Measure	Experimental Group (n = 29)	Control Group (n = 29)	Statistical Value	*p*-Value
Age (years)	53.66 ± 9.12	55.21 ± 8.76	t = −0.66	*p* > 0.05
Gender (Male/Female)	16/13	14/15	χ^2^ = 0.276	*p* > 0.05
FEV1/FVC (%)	74.48 ± 4.75	76.08 ± 4.51	t = −1.31	*p* > 0.05

Note: FEV1/FVC, the ratio of forced expiratory volume in 1 s to forced vital capacity, is a standard indicator of pulmonary function used to assess airflow limitation.

**Table 3:** Comparison of primary observation indicators between the two groups.

Outcome Measure	Experimental Group (n = 29)	Control Group (n = 29)	Statistical Value	*p*-Value
Image retrieval time (s)	75.16 ± 19.38	209.59 ± 28.13	t = −21.19	*p* < 0.001
Total operation time (min)	88.72 ± 13.82	96.55 ± 13.90	t = −2.15	*p* = 0.036
Intraoperative blood loss (mL)	26.21 ± 12.37	29.31 ± 14.13	t = −0.89	*p* = 0.38

**Table 4:** Comparison of secondary observation indicators between the two groups.

Outcome Measure	Experimental Group (n = 29)	Control Group (n = 29)	Statistical Value	*p*-Value
R0 resection rate (%)	100	100	-	-
Postoperative drainage time (days)	2.41 ± 0.73	2.28 ± 0.53	t = 0.82	*p* = 0.41

## Data Availability

The data presented in this study are available on reasonable request from the corresponding author due to privacy concerns.

## Abbreviations

The following abbreviations are used in this manuscript:

NSCLC	Non-small cell lung cancer
CNN	Convolutional neural network
TBD	Track before detect
FEV1	Forced expiratory volume in 1 s
FVC	Forced vital capacity
